# Emerging Factors Implicated in Fibrotic Organ–Associated Thrombosis: The Case of Two Organs

**DOI:** 10.1055/s-0039-1692204

**Published:** 2019-06-07

**Authors:** Orly Leiva, Roelof H. Bekendam, Brenda D. Garcia, Cristal Thompson, Alan Cantor, Vipul Chitalia, Katya Ravid

**Affiliations:** 1 Department of Medicine, Boston University School of Medicine, Boston, Massachusetts, United States; 2 Department of Medicine, Brigham and Women's Hospital and Harvard Medical School, Boston, Massachusetts, United States; 3 Department of Medicine, Mount Auburn Hospital and Harvard Medical School, Boston, Massachusetts, United States; 4 Whitaker Cardiovascular Institute, Boston University School of Medicine, Boston, Massachusetts, United States; 5 Children's Hospital Boston, Boston, Massachusetts, United States; 6 VA Boston Healthcare System, Boston, Massachusetts, United States

**Keywords:** thrombosis, myelofibrosis, chronic kidney disease, lysyl oxidase, fibrosis

## Abstract

Thrombosis is at the heart of cardiovascular complications observed in specific diseases. A heightened thrombosis risk above that in general population in diseases such as myelofibrosis and chronic kidney disease implicates disease-specific mediators of thrombosis. This relative lack of information regarding the mechanisms of thrombosis in specific organ pathologies hitherto has remained limited. Evolving literature implicates some soluble factors in the blood of patients with discrete disorders, inflicting fundamental changes in the components of thrombosis. In this era of precision medicine, integrating these disease-specific factors in a comprehensive thrombotic risk assessment of patients is imperative in guiding therapeutic decisions. A complex network of mechanisms regulates each organ pathology and resultant thrombotic phenotypes. This review surveys different effectors of thrombogenicity associated with two pathologically fibrotic organs used as model systems, the bone marrow and kidney, as well as focuses attention to a common inducer of fibrosis and thrombosis, lysyl oxidase.

## Overview

Cardiovascular disease has protean manifestation including stroke and acute coronary syndromes (ACSs), heart failure, and venous thrombosis with pulmonary embolism (combined as venous thromboembolism [VTE]). Arterial or venous or microvascular thrombosis underlies most of these events either as a direct causal factor (stroke or ACS or VTE) or as an important contributor. While cardiovascular disease remains a number 1 cause of death in general population, its risk is increased in certain unrelated diseases such as primary myelofibrosis (PMF), chronic kidney disease (CKD), or cancer, to name a few.

PMF, which is characterized by augmented proliferation of cells of the myeloid lineage, the megakaryocytes, and a fibrotic marrow, 1 is associated with increased propensity for cardiovascular disease. 2 3 A study of 707 patients with PMF followed up in four European institutions showed that fatal and nonfatal thromboses were documented in 51 (7.2%) patients, with a rate of 1.75% patient-years. Of patients with nonfatal cardiovascular events (47), 1% had acute myocardial infarction (MI) and 3.1% had VTE. 4 The risk of developing a fatal or nonfatal thrombotic event in PMF was found to be 2.2% patient-years. PMF has been associated with increased risk of both venous and arterial thrombosis. 5 6 A very recent meta-analysis estimated that the overall prevalence of thrombosis in patients with myeloproliferative neoplasms (MPNs) is 20% with the prevalence of arterial thrombosis (cerebrovascular disease, transient ischemic attack, coronary artery disease, and peripheral artery disease) being 16.2% and VTE being 6.2%. 7 Another meta-analysis led to the conclusion that JAK2V617F mutation in PMF patients is associated with an increased risk of thrombosis (odds ratio: 1.76, 95% confidence interval [CI]: 0.91–3.41). 4

As for CKD, currently approximately 10% of the adult population in the United States and worldwide suffer from this pathology. These rates are rising at an alarming proportion, and CKD patients will be 28 million in 2020 and nearly 38 million in 2030 in the United States. 8 9 Similar to PMF, end-stage renal disease is associated with a 2.3-fold increase risk of VTE as compared with the general population, 10 and patients on dialysis have 11.9-fold and 8.4-fold increased chance of developing ACS and stroke, respectively. 11

This substantial increase in the risk of cardiovascular events suggests a possibility of underlying disease-specific mediators. While the general mechanisms of thrombosis have been well defined and have driven the development of current antiplatelet and antithrombotic agents, the disease-specific factors that augment thrombotic risk in each pathology remain less characterized. It is important to investigate the disease-specific mediators to develop biomarkers or therapeutic targets to augment the efficiency of current antithrombotic that largely perturb normal hemostatic defense in the blood. Accordingly, the aim of this review is to focus on two organ systems as means of illustrating specific organ pathology-evoked mediators of thrombosis. More specifically, the goal is to shed light on various PMF- or CKD-associated factors that are involved in the pathophysiology of their respective diseases but also contribute to increased thrombotic risk.

## From Pathological Fibrosis in Primary Myelofibrosis to Thrombosis

PMF is the least frequent among the MPNs. It can range from pre-PMF, exhibiting JAK2, CALR, MPL mutations, megakaryocyte proliferation, and atypia with grade 1 fibrosis, to overt PMF, which displays grades 2 to 3 fibrosis. 1 12 As noted earlier, human studies suggest that JAK2V617F mutations in PMF are associated with higher rates of thrombosis, and increased platelet activation, with a greater allele burden portending the highest risk. 13 14 Mimicking human phenotypes in mice has uncovered an interesting interplay of different components driving thrombosis in various types of MPN. For example, models using mainly polycythemia vera/PMF phenotype showed highly unstable thrombi in a ferric chloride-induced injury model of thrombosis and prolonged bleeding times, compared with matching controls. 15 In other systems, where the phenotype was more essential thrombocytosis (ET)-like, increased platelet reactivity to some agonists was found with decreased thrombosis after injury driven by an acquired von Willebrand factor (vWF) deficiency. 16

### Platelets in Primary Myelofibrosis

Platelets constitute a critical component of thrombus formation and propagation. 17 18 Upon exposure to specific ligands, platelets undergo rapid activation that leads to platelet adhesion, aggregation, and secretion of granule content. Platelets can be activated by circulating factors, such as thrombin, adenosine diphosphate (ADP), and epinephrine, which target specific receptors. 19 20 Platelets are also activated upon interaction with subendothelial collagen and fibronectin exposed in an injured vasculature. 21 22 Platelet binding to the vessel wall is mediated through the collagen receptors, integrins α2β1 or glycoprotein (GP)VI, 23 or the fibronectin receptors αvβ3 or αvβ1, or indirectly through vWF via platelet GPIb/IX/V complex. 24 25 Type IV collagen is the only matrix protein that supports both platelet adhesion and complete activation. 23 Epidemiologic data revealed a potentially important role for α _2_ β _1_ in thrombotic events, 26 27 28 29 30 31 and higher expression of GPVI is associated with poorer outcome in ACS. 32 Although some of these receptors might play a role in heightened platelet activation documented in PMF patients, 33 antiplatelet therapy is currently not part of a routine regimen of treatment. While a study showed that the effectiveness of aspirin therapy in PMF patients depends on platelet count, 34 randomized data with regard to antiplatelet therapy remains undocumented.

### Contribution of Vascular and Immune Cells to Thrombogenicity in PMF

PMF is also associated with changes in cells that are known to contribute to a thrombotic phenotype, such as vascular or immune cells. The contribution of endothelial cell dysfunction in MPN was established using mice carrying specific JAK2V617F knock-in mutations. These mice have defective platelet responses to ristocetin, with unaltered platelet counts, suggesting a role for JAK2V617F-mediated vWF processing. 16 This implicates that endothelial dysfunction, with decreased vWF secretion and processing, is a contributing factor to the JAK2V617F phenotype. Furthermore, endothelial cells carrying the JAK2V617F mutation display prothrombotic features, 35 and vascular endothelial cell expression of JAK2V617F promote **s** thrombosis owed by upregulated P-selectin. 36 Patients with JAK2V617F-mutated MPN were shown to have increased megakaryocyte heparanase expression, possibly through an upregulated erythropoietin receptor. 37 Heparanase is an upregulator of tissue factor (TF) in endothelial cells through a p38-mediated phosphorylation and subsequent increase in procoagulant activity. This may contribute to hypercoagulability in PMF. 37 38 Recently, granulocytes in JAK2V617F PMF mice have also been shown to contribute to the increased thrombogenic phenotype in mice that underwent inferior vena cava partial ligation. 39 Anti-α4-integrin (anti-VLA-4) and anti–β2 integrin antibodies targeting granulocytes interfered with thrombosis through inhibition of leukocyte–endothelial interactions. Additionally, abnormal trafficking of JAK2V617F-positive granulocytes to the white pulp and marginal zone of the spleen could be prevented by the use of anti–β2 antibodies. 39 Alterations in other soluble factors in blood and in the vessel wall matrix in PMF could augment their thrombogenicity. PMF is associated with elevation of interleukin (IL)-6 and C-reactive protein (CRP) which are known to contribute to increased risk for thrombotic events. Disease progression correlates with higher CRP levels and higher JAK2V617F levels. 40 Finally, hypermethylation of MAC1, a receptor on leukocytes primarily involved in phagocytosis, was found to be an independent risk factor for thrombotic complications in patients with MPN. 41 Increased expression of MAC-1 on neutrophils allows interactions with platelets to assist in the assembly of coagulation proteins. Thus, several factors in PMF contribute to platelet or vascular activation, the level and significance of which would require personalized assessment.

### The Role of a Fibrosis Regulator, Lysyl Oxidase

Though the earlier-described cell types have been implicated in promoting a thrombotic phenotype in PMF, lysyl oxidase (LOX) is an enzyme secreted from megakaryocytes in PMF, with ability to control both fibrosis and thrombosis. LOX catalyzes the final reaction required for biosynthetic collagen and elastin cross-linking and maturation to result in functional connective tissues. LOX is elevated in megakaryocytes of JAK2V617F PMF mice and human samples, and may contribute to the thrombophilia in these patients. 42 43 LOX is selectively expressed in 2N and 4N megakaryocytes. LOX expression was approximately 1.5-fold higher in PMF megakaryocytes. Interestingly, LOX expression was approximately 20-fold higher in platelets when compared with healthy subjects. 44 In addition to the increased blood levels, celltype specific increase in LOX also contributes to the thrombosis. Human PMF platelets were also found to have elevated LOX and increased α2β1-mediated platelet adhesion to collagen. 42 Increased LOX in platelets led to augmented platelet aggregation in response to collagen and quicker time to thrombosis in a carotid artery injury model of thrombosis. 45 Using LOX pharmacological inhibitor, this study concluded that the collagen receptor α2β1 is regulated by LOX. Transgenic upregulation of LOX in WT mice resulted in increased interaction between monomeric collagen and the α2β1 integrin. 45 On the other hand, fibrillar collagen did not enhance adhesion in both transgenic-upregulated LOX-containing platelets and PMF platelets.

## From Chronic Kidney Disease and Fibrosis to Thrombosis

As noted earlier, CKD is a strong and independent risk factor for cardiovascular disease. 46 CKD is marked by loss of function of glomeruli, and by gradual development of fibrotic tissue. 47 Fibrosis is a common final mechanism of CKD irrespective of inciting pathology initiating CKD. Decreased renal function is a risk factor for death after MI and percutaneous coronary intervention (PCI). 48

### The Role of Endothelial Cells and Platelets

Several components of thrombosis are affected in CKD. Patients with CKD have increased platelet aggregation with ADP at baseline than non-CKD patients. 49 50 The endothelium has also been recognized as a significant contributor to the prothrombotic state in CKD. Endothelial vWF and plasminogen activator inhibitor-1 secretion in patients with CKD is increased. Next to this, several prothrombotic coagulation factors (VII, VIII, IX–XII) show increased activity in CKD, while protein C level is reduced. Further, platelets become increasingly responsive to agonists. Second, the endothelium loses its ability to maintain vascular quiescence by decreased nitric oxide bioavailability through increase in oxidative stress, and upregulation of procoagulant prostaglandins through endothelial COX-2. Third, uremic toxins lead to overexpression of TF in monocytes and endothelial cells, further propagating a prothrombotic phenotype. 51 52 Despite a hyperthrombotic phenotype, not all CKD patients respond effectively to clopidogrel, an inhibitor of platelet ADP receptor, and a commonly used antiplatelet medication. 49 50 53 54 This is important since poor response to clopidogrel has been associated with increased risk of death, MI, and stent thrombosis in patients undergoing PCI. 55 The antiplatelet effects of aspirin have also been reported to be reduced in patients with CKD, 56 although some studies reported no difference in platelet aggregation in CKD patients compared with non-CKD. 49 While it is currently unknown why some CKD patients do not experience reduced thrombotic risk upon clopidogrel administration, studies suggest that CKD resultant uremia can alter platelet function. 57

### Tryptophan Metabolites and Lysyl Oxidase

CKD is characterized by the accumulation of toxic metabolites due to inability of the kidneys to excrete them properly. Tryptophan is metabolized by bacteria in the intestine to indoles and is further metabolized in the liver to derivatives, such as indoxyl sulfate (IS), that accumulate in patients with CKD, unless dialyzed. 58 Several studies have shown IS to be damaging to endothelial cells, and to induce platelet hyperactivity which may play a role in the risk of thrombosis in CKD. 57 59 60 61 In a CKD mouse model, IS was found to induce platelet hyperactivity largely due to reactive oxygen species (ROS) production. 59 Another mechanism by which IS may facilitate thrombosis in CKD is by upregulating the expression of TF in vascular tissue via the aryl hydrocarbon receptor (ARH). 57 62 On the other hand, augmented TF is a known risk factor for cardiovascular disease. 63 64 IS also contributes to cardiovascular disease by promoting the development of atherosclerosis. 65 IS induces phosphorylation of platelet-derived growth factor (PDGF)-β receptors on vascular smooth muscle cells (VSMCs), which leads to the activation of the receptor and subsequent migration and proliferation. 65 Furthermore, IS upregulates the expression of the PDGF-β receptor and sensitizes it to PDGF by generation of ROS through NADPH oxidase-dependent mechanisms.

Another tryptophan metabolite that accumulates in CKD patients is kynurenine (KYN). 66 The rate-limiting enzyme responsible for the degradation of tryptophan to KYN is indoleamine 2,3-dioxygenase 1 (IDO1). 67 Activity of IDO1, as measured by the ratio of KYN:tryptophan, has been associated with progression of CVD and coronary heart disease. 68 69 Patients with CKD have increased activity of IDO1. 70 71 72 One study found a correlation between IDO1 activity and increased carotid artery atherosclerotic plaque size and decreased ankle–brachial index (a marker of peripheral vascular disease) in patients with CKD. 70 A study of 473 patients with advanced CKD demonstrated that patients with subsequent arteriovenous fistula thrombosis had higher levels of KYN compared with those without thrombosis. KYN elevates TF levels in cultured smooth muscle cells. 61 Also of note, KYN plasma levels were reported to predict acute coronary events. 73 Finally, as mentioned earlier, fibrosis is part of CKD progression. In a model of diabetic nephropathy, there is increased LOX expression in the glomerular and tubular areas of the nephron. 74 75 It is then conceivable that in CKD too, LOX contributes to altered platelet properties, in addition to its effect of tissue fibrosis.

## Conclusions and Implication for New Therapeutics

Although we focused on two fibrotic organs as model systems of pathology-induced thrombosis, there are several other examples that follow a similar paradigm. For instance, systemic sclerosis (SSc) is an autoimmune disorder characterized by immune activation, leading to skin and visceral organ fibrosis, 76 with cardiovascular disease and stroke being the most common cause of death. 77 78 79 Platelets in SSc patients have increased platelet responsiveness to ADP, serotonin, and collagen, 80 81 82 and just as in PMF, tissue and serum LOX is elevated in SSc patients. 83 84 85 Therefore, understanding mechanisms of newly identified factors that impact platelet and vascular function in different pathological conditions, and exploring consequences of their pharmacological modulation will offer a window to specific prognostic biomarkers that will (1) advance future development of therapies that target disease-induced atherothrombotic disorders and (2) encourage the general concept of integrating myelofibrosis and other fibrotic organ–specific factors in the global thrombotic and cardiovascular risk assessment. The contribution of nutrition through a low-tryptophan–based diet has the theoretical potential to alter the indole solute-associated risk of thrombotic complications in patients with CKD. Upregulation of LOX in fibrotic organs serves as a potential target for antithrombotic therapy. For instance, it would be interesting to examine the role of LOX in the thrombogenic tendency in a mouse experimental model of CKD. Further, more extensive mechanistic studies are needed in each case to advance disease-specific therapeutic development and personalized care to patients suffering from a variety of thrombogenic pathologies.
